# The Case Selection for Vaginal Cuff Brachytherapy in Cervical Cancer Patients After Radical Hysterectomy and External Beam Radiation Therapy

**DOI:** 10.3389/fonc.2021.685972

**Published:** 2021-06-25

**Authors:** Ying-Lu Lai, Ye-Ning Jin, Xi Wang, Wei-Xiang Qi, Rong Cai, Hao-Ping Xu

**Affiliations:** ^1^ Department of Radiation Oncology, Ruijin Hospital, Shanghai Jiao Tong University, School of Medicine, Shanghai, China; ^2^ Department of Oncology, Zhejiang Xiaoshan Hospital, Zhejiang, China; ^3^ Department of Neurology, Hackensack Meridian Health JFK Medical Center, Edison, NJ, United States

**Keywords:** cervical cancer, post-hysterectomy, adjuvant radiotherapy, brachytherapy, case selection

## Abstract

**Objective:**

To explore the suitable cases for vaginal cuff brachytherapy (VCB) combined with external beam radiation therapy (EBRT) in the postoperative treatment of cervical cancer.

**Methods:**

We retrospectively analyzed the clinical data of 214 postoperative cervical cancer patients who received radiotherapy from January 2008 to December 2015. Among them, 146 patients received postoperative EBRT, 68 received EBRT plus VCB. There was no statistical difference in clinical and pathological characteristics between these two groups. Those who with negative vaginal cuff underwent supplemented 12–18 Gy/2–3 Fx VCB. Survival analyses were performed using Kaplan–Meier method, and Cox model was used to analyze prognostic factors.

**Results:**

The median follow-up was 52 months (9–136 months), and 4-year RFS (recurrence-free survival) was 77%. Among them, 58 patients (27.10%) had local or distant recurrences, 29 (13.55%) in pelvis, six (2.80%) with metastases to para-aortic, 19 (8.88%) with distant metastases (including inguinal lymph nodes) and four (1.87%) with both local and distant recurrences. The postoperative brachytherapy boost did not improve RFS or OS (overall survival) among the investigated subjects, *P* = 0.77, *P* = 0.99, respectively. Neither it decreased the local relapse in the pelvis or vaginal cuff, *P* = 0.56, *P* = 0.59. Subgroup analyses showed that brachytherapy boost improved RFS in patients who had bulky mass (>4 cm) as well as 1) with deep stromal invasion (>50% stromal invasion), *P* = 0.012 or 2) received low EBRT dose (≤45 Gy), *P* = 0.033, and in patients with deep stromal invasion as well as received low EBRT dose (*P* = 0.018).

**Conclusions:**

We first proposed the case selection model for postoperative EBRT plus VCB. Brachytherapy boost were considered in the setting of postoperative radiotherapy if the patients had at least two out of these following factors: bulky mass, deep stromal invasion and low EBRT dose.

## Introduction

Early-stage carcinoma of uterus cervix has a relatively favorable prognosis. Certain clinical and pathologic risk factors for the recurrences have been identified. The randomized trial GOG 92 (1) has shown the benefit of adjuvant external beam irradiation in the early-stage patients with negative lymph node. At 2 years’ follow-up, the recurrence-free survival was 88% for adjuvant RT versus 79% for the no-adjuvant RT group. A clear trend towards improving OS was noted after long-term (12 years) follow-up, 67% *vs.* 40% (*P* = 0.07). According to the results of GOG 92, the “Sedlis Criteria” (2) was used to guide adjuvant radiotherapy in patients with LVSI, deep stromal invasion or bulky mass, which were considered as the intermediate risk factors of recurrences. However, we found that brachytherapy was not included in the GOG 92, and local recurrence in this study was the main pattern of failure, both in the external beam irradiation group and in the no further treatment group, 18/21 in EBRT arm and 27/39 in no EBRT arm. A relatively high local recurrence remains a problem in early cervical cancer patients whether or not they have received EBRT (without brachytherapy) postoperatively when they have certain risk factors.

Nevertheless, the role of adjuvant brachytherapy boost remains uncertain. Guidelines from the American Brachytherapy Society recommended postoperative adjuvant brachytherapy for non-radical surgery, close or positive margins, large or deeply invasive tumors, parametrial or vaginal involvement, or extensive LVSI. On the other hand, the guideline also mentions that there is no final conclusion on whether or not the postoperative brachytherapy should be used, and the dose is controversial (3).

Many studies supported the use of postoperative radiotherapy, but brachytherapy is not always included. Thus the role of brachytherapy as a supplement to postoperative EBRT remains unclear, particularly in the absence of randomized prospective trials to address this question. Evidence to support the use of brachytherapy comes from a Chinese retrospective study (4), which assessed the progression-free survival and survival outcomes in 113 cervical cancer patients with node-positive IB1–IIA2 stage receiving postoperative EBRT with or without vaginal brachytherapy. They found that the patients in pelvic EBRT with brachytherapy group had a significantly improved 5-year progression-free survival rate (*P* = 0.044), although no significant difference in 5-year overall survival was found between these two groups (*P* = 0.437).

Another retrospective study (5) investigating 79 patients with high-risk early-stage operable cervical cancer, revealed that vaginal cuff brachytherapy boost was associated with a reduced recurrence rate in the postoperative setting. However, they placed more emphasis on the patients with relatively poor prognosis among early-stage population, which were more often presumed as candidates for the definite chemoradiation therapy, especially with the changes of FIGO staging.

This study was designed to evaluate the role of adding vaginal cuff brachytherapy to postoperative EBRT in early-stage cervical cancer patients, especially in those who with the pathologic “intermediate-risk” factors.

## Methods and Materials

### Patients

Some 276 patients with operable early cervical carcinoma treated with postoperative radiotherapy were documented at the radiation oncology department of Ruijin Hospital from January 2005 to December 2015. All patients underwent a complete pretreatment staging workup. The inclusion criteria of this retrospective study were as follows: a) patients underwent radical hysterectomy and pelvic lymphadenectomy, pathologically proven squamous cell carcinoma, adenocarcinoma, or adenosquamous carcinoma of the cervix, without evidence of distant metastasis at diagnosis; b) no concurrent or previous malignant disease, without received previous radiotherapy; c) WHO performance status of 1 or less; d) feasibility of radiotherapy and chemotherapy if it is needed; e) and those who were lost to follow-up were excluded.

All hospital charts and radiotherapy records were reviewed and a total of 214 eligible patients in our cancer registration database were included in this study. Tumor staging was defined according to the International Federation of Obstetrics and Gynecology (FIGO, 2009) staging system. Written informed consent was obtained from each patient before treatment. The patient information was anonymized and de-identified prior to analysis. The study was approved by the local ethics committee of Ruijin Hospital, and it was performed under the ethical standards of the Helsinki Declaration 1975, revised in 1983.

### Radiotherapy and Concurrent Chemotherapy

All of the patients enrolled in this study for the adjuvant EBRT met the Sedlis criteria (2). Those who met the Peters criteria (6) in the study were given concurrent chemotherapy. Radiotherapy was delivered using Intensity Modulated Radiation Therapy (IMRT) technique with seven gantry positions, three-dimensional conformal radiation therapy (3D-CRT) or conventional radiation therapy. Treatment planning CT scans with treatment planning dose information were demanded for both IMRT and 3D-CRT. The CT scans were performed from at least L3 to mid femur. A megavoltage beam of 6 MV or greater were used, with a source-axis distance of 100 cm. For the conventional RT, 4-field technique was applied with CT-based treatment planning. For the IMRT, megavoltage equipment capable of delivering static intensity modulation with a multileaf collimator was used. And the clinical target volume (CTV) of IMRT was contoured according to the Radiation Therapy Oncology Group (RTOG) consensus (7). Planning target volume (PTV) with a 0.7–1 cm margin was given to the CTV uniformly. The prescribed dose was 45 to 50.4 Gy in 25 to 28 fractions (1.8–2.0 Gy/fraction), once per day, 5 days per week. Complete blood count test was performed weekly.

The attending physicians made the treatment decisions. The vaginal cylinder or ovoids was used for brachytherapy as a boost to vaginal cuff. OARs (rectum, sigmoid, and bladder) were contoured according to GEC-ESTRO guidelines (8, 9). The patients with negative vaginal cuff margin received a HDR (high dose rate) brachytherapy using an iridium-192 source, delivered in 2–3 fractions, 6 Gy per fraction, while the patients with positive margin received 6 Gy × 5 or 8 Gy × 3 fractions. The prescription was to the 5 mm below the vaginal surface and the treatment length was upper 3 cm of the vaginal cuff. The total dose achieved 70–80 Gy (EQD2, biologically equivalent dose of 2 Gy per fraction) with negative margin, otherwise it would achieve 85 Gy with close or positive margin. The attending physicians decided whether the patients would receive further VCB after EBRT.

The concurrent chemotherapy was cisplatin 40 mg/m^2^ (maximum of five courses) weekly or paclitaxel 175 mg/m^2^ and cisplatin75 mg/m^2^ (maximum of two courses) every 21 days.

### Follow-Up

After completion of the entire treatment plan, surveillances consisted of a) physical examination, vaginal cytology, abdominal ultrasound and pelvic computed tomography (CT) or Magnetic Resonance Imaging (MRI) every 3 months for the first 2 years, then every 6 months for another year, and then annually; b) chest X-ray or CT annually; c) laboratory assessment and ^18^F-FDG PET-CT imaging were indicated based on symptoms of examination findings suspicious for recurrence. The laboratory assessment included complete blood count (CBC), blood urea nitrogen (BUN) and SCC-Ag (squamous cell carcinoma antigen) for squamous carcinoma, etc. Acute toxicity was assessed weekly during radiotherapy. Toxicity was scored using RTOG criteria (10). Late toxicity was defined as toxicity occurring greater than 90 days after radiation therapy. Grade 2 small/large intestine-late toxicity was defined as moderate diarrhea and colic, bowel movement >5× daily, excessive rectal mucus or intermittent bleeding. Grade 3 small/large intestine-late toxicity was defined as obstruction or bleeding requiring surgery.

### Statistical Analysis

The chi-square test was used to determine the difference of the patients’ characteristics between two subgroups. The sites of relapse were identified as vaginal cuff, pelvis (not including vaginal cuff), para-aortic lymph nodes, inguinal lymph nodes, and other distant. The “bulky mass” in cervical cancer refers to the mass equal or more than 4 cm in greatest dimension. The LR (local recurrences) were categorized as either vaginal cuff recurrence, or recurrence in other parts of pelvis. OS (overall survival) was defined as the time from the start of treatment until the date of death from any cause, and RFS (recurrence-free survival) was measured from the date of the treatment to the date of any recurrence (local or distant) or to the date of death due to any cause. LR (local recurrence) included the recurrence in the vaginal cuff and LRR (loco-regional recurrence) within the pelvis. Data on patients who were alive or without progression were censored at the time of the last follow-up. OS and RFS curves were estimated with the Kaplan–Meier method. And univariate analyses comparing RFS or OS between the two groups were performed by Log-Rank test. The Cox regression models were used to evaluate the difference between the two groups, adjust for prognostic factors, and estimate the relative likelihood of OS and RFS. Data were analyzed by SPSS 20.0 (IBM SPSS Statistics for Windows, Version 20.0; IBM Corp, Armonk, NY), and P <0.05 was considered statistically significant.

## Results

### Patient Characteristics

Some 214 patients (median age 50 years, ranging from 26 to 78 years) with cervical cancer met selection criteria were analyzed from January 2005 to December 2015. Among them, 68 patients received postoperative EBRT followed by vaginal cuff brachytherapy boost, and 146 patients received only postoperative EBRT. Patient demographic and baseline disease characteristics are shown in **Table 1**. Among patients with positive margins, the proportion of patients receiving EBRT + VCB treatment was significantly higher than that of patients receiving external beam radiation EBRT treatment only, *P <*0.001. The median dose to the pelvis of EBRT group was 50 Gy (ranging from 40 to 50.4 Gy, mean dose 47.5 Gy), while the median dose of EBRT + VCB group was 46 Gy (ranging from 40 to 50.4 Gy, mean dose 47.6 Gy). Patients treated before the year 2008 (16 patients) received EBRT only. The median number of cycles for the adjuvant chemotherapy and concurrent chemotherapy was 3 and 4 respectively.

**Table 1:** Patients’ Clinical and Pathological Characteristics.

	N	EBRT only	EBRT + Brachytherapy	*P*
**Age, y**				
≤45	72	49 (68.06%)	23 (31.94%)	1.00
>45	142	97 (68.31%)	45 (31.69%)	
**Stage**				
IA–IB	77	56 (72.73%)	21 (27.27%)	0.49
IIA	104	67 (64.42%)	37 (35.58%)	
IIB–IIIA	33	23 (69.70%)	10 (30.30%)	
**Histology**				
Squamous	180	122 (67.78%)	58 (32.22%)	0.90
Adeno-squamous carcinoma	9	7 (77.78%)	2 (22.22%)	
Adenocarcinoma	25	17 (68.00%)	8 (32.00%)	
**Tumor diameter, cm**				
<4	140	96 (68.57%)	44 (31.43%)	0.88
≥4	74	50 (67.57%)	24 (32.43%)	
**PLN metastases**				
No	124	90 (72.58%)	34 (27.42%)	0.14
Yes	90	56 (62.22%)	34 (37.78%)	
**Deep stromal invasion**				
<1/2	94	61 (64.89%)	33 (35.11%)	0.35
≥1/2	120	85 (70.83%)	35 (29.17%)	
**LVSI**				
No	130	90 (69.23%)	40 (30.77%)	0.89
Yes	82	56 (68.29%)	26 (31.71%)	
unknown	2	0 (0.00%)	2 (100.00%)	
**Parametrial involvement**				
No	186	129 (69.35%)	57 (30.65%)	0.39
Yes	28	17 (60.71%)	11 (39.29%)	
**Positive margin**				
No	199	142 (71.36%)	57 (28.64%)	<0.001
Yes	15	4 (26.67%)	11 (73.33%)	
**Radiotherapy**				
Conventional (2D) radiotherapy	14	8 (57.14%)	6 (42.86%)	0.69
3D-CRT	55	38 (69.09%)	17 (30.91%)	
IMRT	145	100 (68.97%)	45 (31.03%)	
**Chemotherapy**				
Yes	163	108 (66.26%)	55 (33.74%)	0.31
No	51	38 (74.51%)	13 (25.49%)	

EBRT, external beam radiation therapy; LVSI, Lymph-vascular space invasion; PLN metastases, pelvic lymph nodes metastases; 3D-CRT, three-dimensional conformal radiation therapy; IMRT, Intensity-modulated radiation therapy.

### Failure Patterns

The median follow-up was 52 months (range 9–136 months). The four-year RFS rate was 77%. Some 58 patients (27.10%) had local or distant relapse. Among these patients, the site of relapse was the pelvis including the vaginal cuff in 29 patients (13.55%), vaginal cuff alone in 16 patients (7.48%), para-aortic lymph nodes in six patients (2.80%), inguinal metastases in two patients (0.93%), other distant sites in 17 patients (7.94%), and both pelvic and distant metastases in four patients (1.87%), respectively. The failure patterns of these two groups were shown in **Table 2**.

**Table 2:** Failure Patterns by Treatment Regimen.

Sites of relapses	Total (%) (N = 214)	EBRT only (%) (N = 146)	EBRT + Brachytherapy (%) (N = 68)	P
**Vaginal cuff**	16 (7.48%)	10 (6.85%)	6 (8.82%)	0.59
**Pelvis (not vaginal cuff)**	13 (6.07%)	8 (5.48%)	5 (7.35%)	0.56
**Para-aortic lymph nodes metastases**	6 (2.80%)	4 (2.74%)	2 (2.94%)	1.00
**Distant metastasis (including Inguinal lymph nodes)**	19 (8.88%)	13 (8.90%)	6 (8.82%)	1.00
**Pelvic and Distant metastases**	4 (1.87%)	3 (2.05%)	1 (1.47%)	0.77

EBRT, external beam radiation therapy; VCB, vaginal cuff brachytherapy.

Subjects with positive pelvic lymph nodes remained the only independent prognostic factors for the relapse sites (*P* = 0.045), indicating patients with positive pelvic lymph nodes had a higher relapse rate in pelvis.

### Surveillance Results

Cox regression model revealed that the patients with pathology of adenocarcinoma cells, advanced FIGO stage, bulky mass (≥4 cm), deep stromal invasion, positive pelvic lymph node, and the dose of EBRT ≤45 Gy were all highly significantly and independently related to risk of recurrences, *P* = 0.017, 0.026, 0.006, 0.025, 0.001 and 0.032. However, adjuvant chemotherapy was not a significant factor that influenced RFS, *P* = 0.70, which did not reduce the risk of pelvic relapse or distant metastasis. Neither did chemotherapy influence the sites of relapse, *P* = 0.48.

Patients did not benefit from the postoperative brachytherapy boost, as the RFS (range 9–101 months in EBRT+VCB group and 3–136 months in EBRT group) and OS (range 12–101 months in EBRT+VCB group and 9–136 months in EBRT group) were similar between the two groups with or without brachytherapy, *P* = 0.77 and 0.99.

### Subgroup Analysis

**Figure 1:**
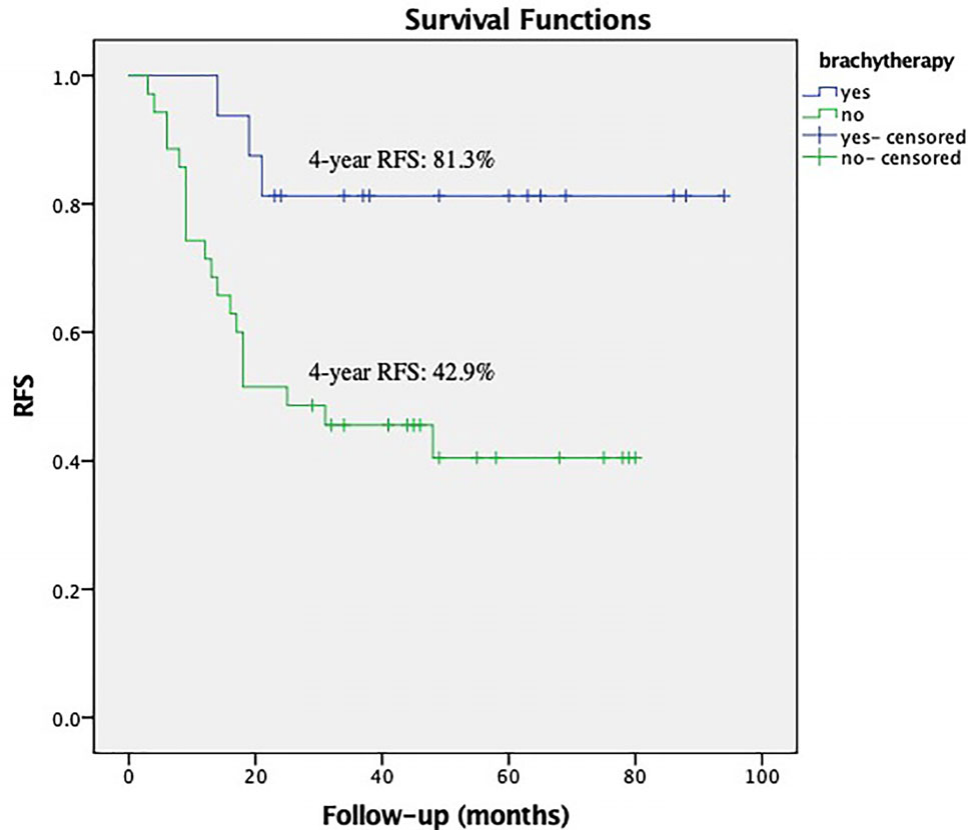
Recurrence Free Survival (RFS) with or without vaginal cuff brachytherapy in patients with a bulky mass and deep stromal invasion. The 4-year RFS was 81.3% *vs.* 42.9%, respectively, *P* = 0.012.

**Figure 2:**
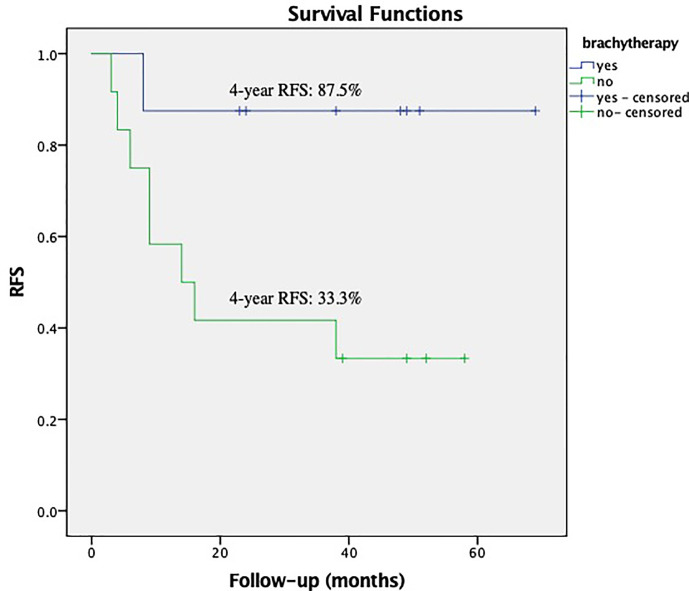
Recurrence Free Survival (RFS) with or without vaginal cuff brachytherapy in patients with a bulky mass that received low external beam radiation therapy dose. The 4-year RFS was 87.5% *vs.* 33.3%, respectively, *P* = 0.033.

Within the subgroup of patients with a bulky mass, the Kaplan–Meier analysis showed that brachytherapy boost improved RFS in those with a deep stromal invasion (the 4-year RFS was 81.3% *vs.* 42.9%, respectively, *P* = 0.012, **Figure 1**) or in those with low EBRT dose (≤45 Gy), the 4-year RFS was 87.5% *vs.* 33.3%, respectively, *P* = 0.033, **Figure 2**. The multivariate survival analysis revealed that the brachytherapy was a significant factor influencing the RFS among the patients with deep stromal invasion (range 14–94 months in EBRT + VCB group *vs.* 3–80 months in EBRT group) or with low EBRT dose (range 8–69 months in EBRT + VCB group *vs.* 3–58 months in EBRT group), *P* = 0.016 (HR = 0.42, 95%CI 0.21–0.85), *P* = 0.048 (HR = 0.12, 95%CI 0.01–0.98).

**Figure 3:**
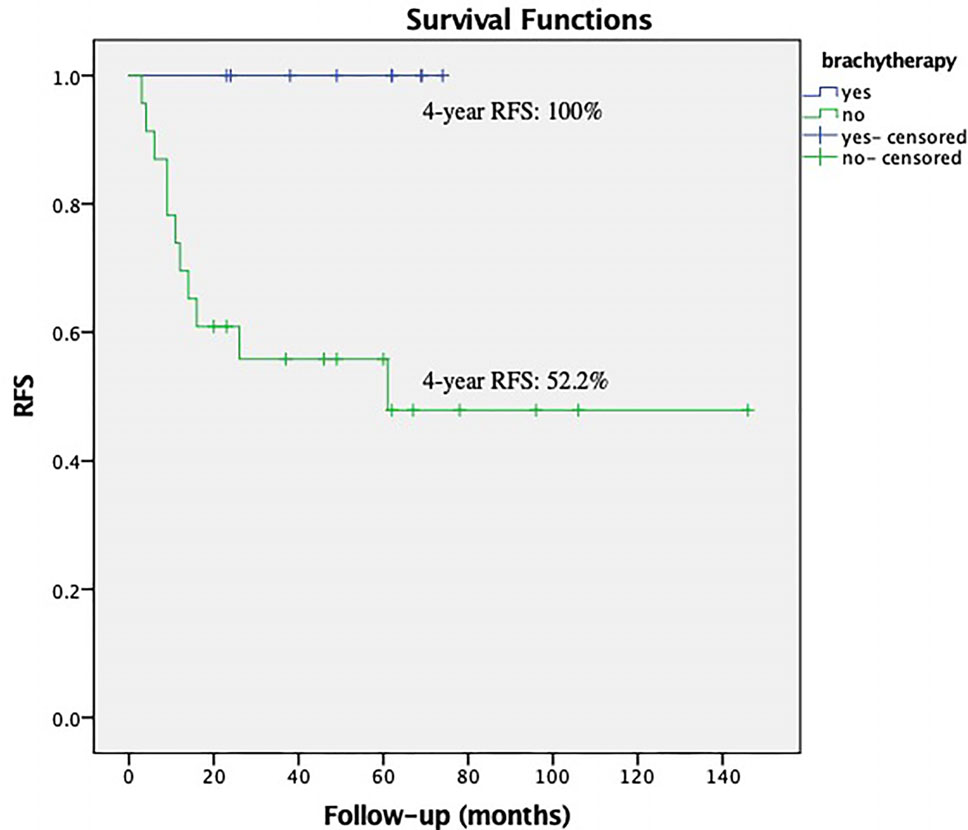
Recurrence Free Survival (RFS) with or without vaginal cuff brachytherapy in patients with deep stromal invasion that received low external beam radiation therapy. The 4-year RFS was 100.0% *vs.* 52.2%, respectively, P = 0.018.

Within the subgroup of patients with low EBRT dose (the patients received EBRT dose ≤45 Gy), the brachytherapy boost significantly reduced the relapses in those with deep stromal invasion, *P* = 0.018 (**Figure 3**). The multivariate survival analysis showed brachytherapy was the independent factor influencing the RFS (range 23–74 months in EBRT + VCB group vs. 3–106 months in EBRT group) among the patients with deep stromal invasion and received low EBRT dose, *P <*0.001 (HR = 0.11, 95%CI 0.04–0.36).

Within the subgroup of patients with positive margins (16 cases), seven out of 11 patients received EBRT + VCB treatment had local or distant relapses including one in vaginal cuff, three cases in pelvis (not vaginal cuff) and three with distant metastases (including inguinal lymph nodes). While one out five patients received EBRT treatment only had local relapse (the vaginal cuff).

### Toxicity

No treatment-related deaths occurred during the course of this study. Hematologic complications were the most frequent complications. Some 18 patients developed Grade 3 neutropenia and/or thrombocytopenia, 11 in EBRT group (11/164, 7.53%) and seven in EBRT + VCB group 7/68,10.29%), however no significant difference was shown between EBRT group and brachytherapy boost group, *P* = 0.60. Four patients (4/68, 5.88%) developed grade 2 late rectal toxicity of intermittent bleeding in brachytherapy boost group.

## Discussion

Our study analyzed the effects of vaginal cuff brachytherapy on RFS in the adjuvant setting for early-stage cervical cancer patients. We present criteria for selecting cases for postoperative adjuvant brachytherapy in addition to EBRT for early cancer of the cervix. Although this was a retrospective analysis, it needs further explanation why patients were treated with EBRT or EBRT + VCB. There were many factors that influenced treatments decisions. 1) The effect of brachytherapy in the settings of post-operative radiotherapy was unclear. 2) Brachytherapy had not started in our center by the year 2008. 3) The attending physicians have slightly different understandings on the settings of postoperative radiotherapy. However we found there was no significant difference in clinical characteristics between the two groups in our study (**Table 1**).

In this study, pathologically positive lymph nodes were detected in 42.1% (90/214) of the patients, presenting with a higher relapse rate in pelvis, *P* = 0.045. Positive lymph nodes have been recognized as the poor prognosis factor (11, 12). Another retrospective study (4) showed that postoperative pelvic EBRT with brachytherapy had a significantly improved 5-year PFS rate (*P* = 0.044) compared to EBRT alone among those with positive lymph nodes patients. The author recommended the combination of concurrent pelvic EBRT and chemotherapy with vaginal brachytherapy for the treatment of pelvic node-positive cervical cancer.

Within the subgroup of patients with positive margins, a small group of 16 patients, most of them received further VCB treatment as the NCCN and ABS guidelines recommended, which decreased the relapse rate in vaginal cuff from 20% (1/5) to 9.10% (1/11) in our study. More pelvic and distant relapses were observed in the patients with positive margins in EBRT + VCB group, which was due to the fact that there existed other risk factors like positive pelvic lymph nodes, bulky mass deep stromal invasion or parametrial involvement in this group of patients. However in a recent study (13), a total of 480 patients were analyze, the authors concluded that the addition of vaginal brachytherapy of adjuvant EBRT with or without chemotherapy has no benefit on local control or survival rates, even in cases with positive surgical margins. However, we found an apparent limitation was that the majority of the patients with positive margin received EBRT + VCB in their study, with only nine patients received EBRT alone. There were differences in the distributions of the patients in these two treatment groups (*P <*0.001), which would be likely to cause deviations of the results. Another analysis of 1,719 subjects in National Cancer Database (NCDB) (14) has demonstrated that the addition of BT to EBRT is associated with improved survival in patients with positive margins after hysterectomy, while the authors considered there was no difference in survival between EBRT + VCB and EBRT alone group in patients with negative margins. This is consistent with what has been found in the total study population analysis of our study. Unfortunately, they didn’t further investigate the other risk factors that might be contributed to the case selection for postoperative EBRT + VCB treatment.

However, the operable patients with pathologic “intermediate risk” factors are our main concern in our study. On one hand, there were not many early-stage cases in this study presented with pathologic high-risk factors, parametrial involvement was present in 28 cases (13.1%) and positive margin in 15 cases (7.0%). On the other hand, more subjects with “high-risk factors” like parametrial invasion would be detected before surgery and be referred to the definite chemoradiation therapy with the development of imaging techniques, as MRI and PET-CT scan were widely applied for preoperative evaluation (15, 16). MR imaging was reported to assess the extent of local tumor with high accuracy, while LN metastasis and deep cervical stromal invasion of cervical cancer could be well predicted before proceeding PET/CT, which was more accurate compared with the traditional preoperative staging system.

One of the risk factors to select patients for postoperative brachytherapy boost in our study was the relatively low EBRT dose (≤45 Gy in 1.8–2 Gy/fraction) they received. According to the NCCN guidelines, a dose of 45–50 Gy in standard fractionation is generally recommended for the postoperative cervical patients. On retrospective review of the GOG 92 study, patients who received EBRT (dose from 46 Gy in 23 fractions to 50.4 Gy in 28 fractions) without brachytherapy boost, turned out to have the favorable result of local control. In another study (17) for postoperative patients, the external beam irradiation dose was much lower, 40–46 Gy in 1.8–2 Gy/fraction, the OR for the 5-year locoregional recurrence risk (LRR) decreased from 2.5 to 1.15 when the vaginal vault brachytherapy boost (10–14 Gy) was given to the patients with high-risk factors, like parametrial invasion, positive margin, or positive lymph node metastasis in the operative specimen. It seemed that the lower EBRT dose without brachytherapy tended to increase local failure, which were consistent with our findings. In this case, brachytherapy boost might compensate for the insufficient EBRT dose so as to reduce the treatment failure.

The other two factors contributing to postoperative brachytherapy boost were bulky mass and deep stromal invasion. The size of mass is one of the key findings for allocating the stage, while the deep stromal invasion is the factor that may be associated with lymph node metastases, as previous reports have shown (18, 19). These two factors were the independent prognostic factors affecting RFS in our study as well. However, it should be noted that this study suggests using the postoperative brachytherapy boost only if the patient met at least two of these three factors, not only one of them.

In regards to late toxicity, most patients presented without long-term complaints except for the four patients treated early in this study who received postoperative brachytherapy boost of 6 Gy × 5 fractions for their positive margins, presenting with rectal intermittent bleeding. We concerned more about the D90 of the target volume since the dose-volume constriction of OARs for brachytherapy was not so clear at that time. When we looked back to the dose/volume to the rectum, we found that the mean D2cc of the rectum was higher in these four patients. Another limitation of this study was the lack of information on sexual dysfunction during the follow-up, as most of Chinese patients avoided answering this question. However, the rate of sexual dysfunction was reported the same between the patients with or without brachytherapy (5). We think further investigations with more details of the late toxicity are needed with patients shifting their focus to the quality of life over time. In addition, longer follow-up periods are necessary with a sufficient number of patients to get more substantial data of treatment failure patterns and survival outcomes, making the case selection model for the postoperative brachytherapy boost more accurate and convincing.

## Conclusion

We propose a case selection model for postoperative brachytherapy boost. Patients that meet any two of the three following criteria might be the candidates for the postoperative brachytherapy: bulky mass, deep stromal invasion and relatively low EBRT dose.

## Data Availability Statement

The raw data supporting the conclusions of this article will be made available by the authors, without undue reservation.

## Ethics Statement

The studies involving human participants were reviewed and approved by the Ethics Committee of Ruijin Hospital. The ethics committee waived the requirement of written informed consent for participation.

## Author Contributions

HX and YJ were involved in design of the work. YL, WQ, and RC were involved in acquisition of data and drafting the initial manuscript. HX, XW, and YJ were the major contributors in revising the manuscript for important intellectual content. YL and HX gave final approval of the version to be published. All authors contributed to the article and approved the submitted version.

## Funding

This study was sponsored by Shanghai Pujiang Program (No. 2019PJD029).

## Conflict of Interest

The authors declare that the research was conducted in the absence of any commercial or financial relationships that could be construed as a potential conflict of interest.

## Abbreviations

VCB, vaginal cuff brachytherapy; EBRT, external beam radiation therapy; RFS, recurrence-free survival; OS, overall survival; OARs, organs at risk; HDR, high dose rate; LR, local recurrences.
